# Case of Hydatids Discharged by Coughing

**Published:** 1785

**Authors:** 


IX. *Caſe of Hydatids diſcharged by coughing. By Mr.* James Johnſon, *Surgeon at Lancaſter.*

MRS. E. Taylor, of Lancaſter, a widow, aged forty-nine years, and of a corpulent habit of body, had, for the ſpace of three or four years, been troubled with pain in her right ſide, and had been told that ſhe was dropſical, and that her liver was affected, when, in September, 1779, after being ſeverely wet, ſhe was firſt attacked with cough and difficulty of breathing. For theſe complaints I adviſed the occaſional uſe of *elixir. paregoric.* and likewiſe *pilul. ſcill. gum. ammoniac.* and other expectorating medicines, but without affording her any conſiderable relief, as her ſhortneſs of breath, attended with an expectoration of gelatinous matter, continued to increaſe during the winter, and in March, 1780, was become

ſo

ſo diſtreſſing, that ſhe conſented to try the effect of a bliſter between her ſhoulders.

About this time ſhe began to cough up hydatids. The firſt time ſhe obſerved any of theſe, there were four adhering to each other, and all of them were burſt. From this period till the latter end of July, ſhe coughed up more or leſs of hydatids every day. Her cough and difficulty of breathing then began to abate, and ſhe felt herſelf ſo much relieved, that, although ſhe ſtill continued, from time to time, to cough up hydatids, ſhe took no more medicines till January, 1783, when her complaints returned with increaſed violence, and ſhe coughed up a great number of hydatids. *Tinct. thebaic.* gum. ammoniac, ſquills, and other remedies were adminiſtered without any permanently good effect, till at length ſhe took an emetic of *ipecacuan.* and *oxymel. ſcill.* which operated with uncommon ſeverity. During its operation ſhe brought up three hydatids, but has voided none ſince, and from that time her health began to mend, and is now ſo well reſtored, that ſhe is become corpulent, is entirely free from the pain in her ſide, and can walk ſeveral miles a day without being much fatigued. She ſtill, however, has the appearance of an affection of the the liver, and I alſo ſuſpect that ſhe has water in her cheſt.

For ſome time after ſhe had recourſe to the emetic; ſhe took half a grain of calomel every night. Whether or not this had any ſhare in her recovery, I will not pretend to determine.

During her illneſs ſhe coughed up ſeveral hundreds of hydatids, moſt of which were burſt, and of theſe many muſt have been as large as a pullet's egg. Thoſe which were not burſt were only of about the ſize of a nutmeg. They were filled with a gelatinous matter, and many of them were tinged with blood.

